# Prediction of liver and lung metastases in patients with early‐onset colorectal cancer by nomograms based on heterogeneous and homogenous risk factors

**DOI:** 10.1002/cam4.6633

**Published:** 2023-10-30

**Authors:** Yimin E, Sizheng Sun, Xiaoyu Fan, Chen Lu, Pengcheng Ji, Yicheng Huang, Jing Sun, Xiaojun Yang, Chunzhao Yu

**Affiliations:** ^1^ Department of General Surgery The Second Affiliated Hospital of Nanjing Medical University Nanjing China; ^2^ Department of General Surgery Sir Run Run Hospital Nanjing Medical University Nanjing China

**Keywords:** early‐onset colorectal cancer, incidence, metastases, nomogram, SEER

## Abstract

**Background:**

Identifying the risk factors for distant metastasis in early‐onset colorectal cancer (EOCRC) is crucial for elucidating its etiology and facilitating preventive treatment. This study aims to characterize the variability in EOCRC incidence and discern both heterogeneous and homogeneous risk factors associated with synchronous liver, lung, and hepato‐lung metastases.

**Methods:**

This study included patients with EOCRC enrolled in the SEER database between 2010 and 2015 and divided patients into three groups by synchronous liver, lung, and hepato‐lung metastases. Each group of patients with different metastasis types was randomly assigned to the development and validation cohort in a ratio of 7:3. Logistic regression was used to analyze the heterogeneous and homogenous risk factors for synchronous liver, lung, and hepato‐lung metastases in the development cohort of patients. Nomograms were built to calculate the risk of metastasis, and the receiver operating characteristic (ROC) curve and calibration curve were used to quantitatively evaluate their performance.

**Results:**

A total of 16,336 eligible patients with EOCRC were included in this study, of which 17.90% (2924/16,336) had distant metastases. The overall incidences of synchronous liver, lung, and hepato‐lung metastases were 11.90% (1921/16,146), 2.42% (390/16,126), and 1.50% (241/16,108), respectively. Positive CEA values before treatment, increased lymphatic metastases, and deeper invasion of intestinal wall were positively correlated with three distant types of metastases. On the contrary, the correlation of age, ethnicity, location of primary tumor, and histologic grade among the three types was inconsistent. The ROC curve and calibration curve proved to have fine performance in predicting distant metastases of EOCRC.

**Conclusions:**

There are significant differences in the incidence of distant metastases in EOCRC, and related risk factors are heterogeneous and homogenous. Although limited risk factors were incorporated in this study, the established nomograms indicated good predictive performance.

## 1 INTRODUCTION

Colorectal cancer (CRC) is the third most common cancer and the second leading cause of cancer‐related deaths worldwide.^1^ In the past few decades, there has been a significant increase in cases of early‐onset colorectal cancer (EOCRC) in the United States and other high‐income countries. EOCRC is defined as occurring in patients younger than 50 years old.^2^, ^3^ This trend sharply contrasts with the steady decline in incidence and related deaths from late‐onset CRC in the past two decades in America and other high‐income countries.^4^ The decrease in late‐onset CRC incidence and related mortality is primarily attributed to screening, followed by surveillance and treatment.^2^ The incidence of EOCRC has increased by 45% in the past 30 years, with a 1.3% increase in mortality each year since 2008. The US Preventive Services Task Force and the American Cancer Society have recommended lowering the screening initiation age to 45 years. However, patients younger than 50 years old are more likely to be uninsured and have lower compliance with screening, even if they have a family history of CRC.^5^ Additionally, half of EOCRC patients are younger than 45 years old and therefore may not participate in screening. The increase in EOCRC has been accompanied by a decrease in late‐onset CRC, with the median age at diagnosis dropping from 72 years in the early 21st century to 66 years currently.^1^ According to estimates, in the next 10 years, 10%–12% of colon cancer and 25% of rectal cancer will be diagnosed in individuals under the age of 50.^2^

Tumor metastasis is the main cause of poor prognosis in CRC patients. Previous studies have indicated that patients with metastatic CRC have a 5‐year survival rate of only 6%, while the 5‐year survival rate for patients with localized CRC is 90%.^6^ Early‐onset colorectal cancer patients are more likely to experience delayed diagnosis and lack awareness of warning signs and symptoms compared to older patients. Younger CRC patients are more often diagnosed at advanced stages, which is associated with increased mortality.^7^, ^8^ The most common metastatic organ of colorectal cancer is the liver, followed by the lung.^9^ Advances in the treatment of metastatic disease in recent decades, including improvements in surgical techniques, development of targeted therapies, and progress in liver metastasis treatment, have significantly improved the survival of patients with distant metastases. Early detection of distant metastasis is crucial for optimizing management and treatment, improving quality of life, and increasing the 5‐year relative survival rate for patients with first‐time diagnosed colorectal cancer. This holds significant clinical significance.

Imaging examinations, such as computed tomography (CT), positron emission tomography/CT (PET/CT), magnetic resonance imaging (MRI), and laboratory tests including serum tumor markers hold significant diagnostic value for detecting metastasis in CRC patients. CT serves as the primary imaging modality for evaluating distant metastasis in CRC patients. However, its sensitivity for detecting colorectal liver metastases ≥1 cm in diameter is only 65%.^10^ Despite advancements in various auxiliary examinations that have improved the prognosis of colorectal cancer patients, there remains a significant number of cases where distant metastasis is detected late. Therefore, identifying independent risk factors for distant metastasis in patients with colorectal cancer can lead to the early identification of high‐risk individuals. Most previous studies have primarily focused on identifying risk factors for distant metastasis in patients with colorectal cancer across all age groups, while less attention has been given to investigating the risk factors and prognostic variables specific to EOCRC patients, particularly those associated with heterogeneous and homogeneous types of distant metastasis.^11^ The prognosis of CRC patients varies based on diverse clinical and pathological factors, particularly in individuals presenting with distant metastasis. However, there is currently a relative lack of research on the incidence of synchronous liver metastases, lung metastases, and hepato‐lung metastases in EOCRC, and the results of these studies remain controversial.^12^, ^13^ Overall, there have been few systematic studies investigating the heterogeneity and homogeneity of risk factors for synchronous liver metastases, lung metastases, and hepato‐lung metastases in EOCRC patients and establishing risk prediction models based on this information. This means that the disparity and probability of organ‐specific metastases cannot be evaluated in EOCRC patients. Nomogram is a convenient, intuitive, and visual risk prediction tool that quantifies risk by integrating and validating several independent risk factors and have shown unique advantages in multiple studies.^14^, ^15^

Therefore, in this study, a large cohort study utilizing data from the Surveillance, Epidemiology, and End Results (SEER) database is used to characterize the incidence and risk factor differences for synchronous liver, lung, and hepato‐lung metastases in EOCRC patients. Nomograms are developed to assist clinicians in predicting the risk of different distant organ metastases. Early identification of metastasis risk factors can help in developing appropriate medical strategies for EOCRC patients and providing targeted treatment.

## 2 METHODS

### 2.1 Population

The data used for population‐based research came from the SEER database, which is an open public database of the National Cancer Institute (NCI). This study selected patients with EOCRC registered in the SEER database from 2010 to 2015. The database includes demographic and pathological characteristics of patients, as well as information on distant metastasis included at the time of the first diagnosis of colorectal cancer, indicating that all distant metastasis cases were synchronous. This study included patients diagnosed with EOCRC between 2010 and 2015, as well as patients with synchronous liver, lung, and hepato‐lung metastases. Cases diagnosed at autopsy or via death certificates, with the age of < 18 or ≥50, pathologically diagnosed as in situ carcinoma or non‐pathological diagnosis, or with an unknown location of primary tumor were not included in subsequent analyses. Patients without information on distant metastases were also excluded. The final study sample included three groups, namely the liver metastases group (*N* = 16,146), the lung metastases group (*N* = 16,126), and the hepato‐lung metastases group (*N* = 16,108). Each group of patients was randomly divided into a development cohort and a validation cohort in a ratio of 7:3. The number of cases in each group after allocation was as follows: liver metastases group (development cohort: *N* = 11,302; validation cohort: *N* = 4844), lung metastases group (development cohort: *N* = 11,288; validation cohort: *N* = 4838), and hepato‐lung metastases group (development cohort: *N* = 11,276; validation cohort: *N* = 4832). The development cohort was used to determine independent risk factors and build models, while the validation cohort was used for internal validation of the models. A case list was generated using SEER*Stat version 8.4.1.

### 2.2 Statistical analysis

Categorical data were presented as numbers and percentages (*N*, %), and quantitative data were presented as means ± standard deviations (SD). Chi‐square tests were used for comparisons of categorical variables. Univariate and multivariate logistic regression models were applied to identify the risk factors related to distant metastasis of EOCRC. Factors with statistically significant differences in the univariate analysis were included in the multivariate analysis. According to the results of multivariate logistic analysis, the intersection of independent risk factors for different types of metastases was identified to assess heterogeneity or homogeneity, and Venn diagrams were used for visualization. We developed predictive diagrams for synchronous liver, lung, and hepato‐lung metastases in EOCRC. The calibration curves (with 1000 bootstrap samples), receiver operating characteristic (ROC) curves, and the area under the curves (AUC) were used to evaluate the predictive efficacy of the nomograms. Statistical analyses were conducted using GraphPad Prism version 9.0 and SPSS version 26.0. The statistical significance level was set at a two‐tailed *p*‐value of less than 0.05. Venn diagrams were generated using Figdraw. The “rms” and “pROC” packages in R version 4.2.1 were used to draw the nomograms and ROC curves, respectively.

## 3 RESULTS

### 3.1 Clinical and demographic characteristics

**FIGURE 1:**
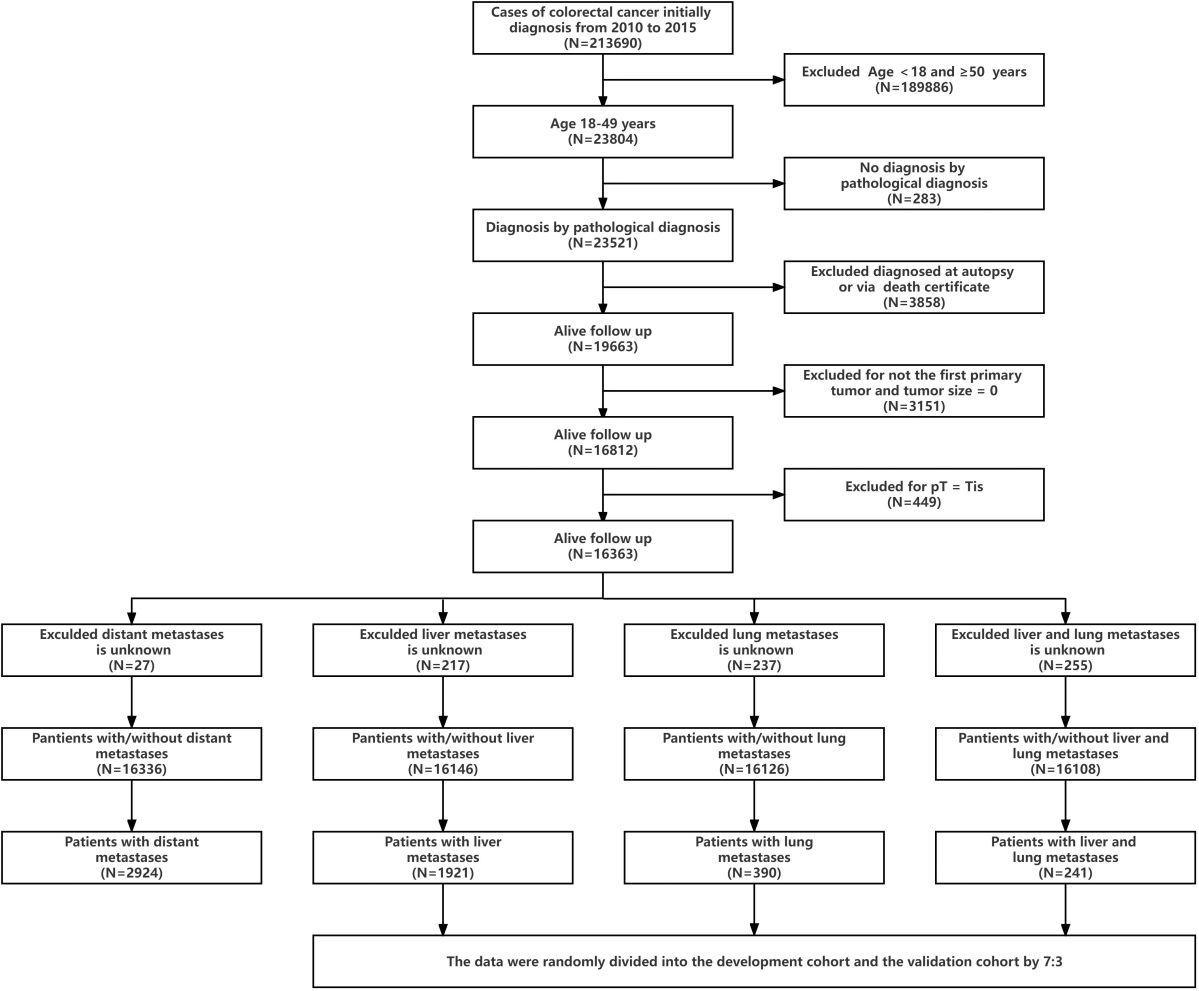
Flowchart of EOCRC patient selection.

A total of 16,363 eligible patients diagnosed with EOCRC between 2010 and 2015 were extracted from the database. The study excluded patients with unknown information regarding distant metastases, resulting in a final sample of 16,336 patients with or without distant metastases. Among these patients, 1921 had liver metastasis only, 390 had lung metastases only, and 241 had both liver and lung metastasis (Figure 1). The average age of the patients was 41.72 ± 6.79 years (range 18–49). 51.23% (*N* = 8369) were male, and 54.9% (*N* = 8973) were married. The majority of patients were white (73.9%, *N* = 12,073). The rectum (28.05%, *N* = 4583) was the most common site of EOCRC. The most prevalent histological type observed was adenocarcinoma, accounting for 60.13% (*N* = 9823), followed by mucinous adenocarcinoma at 7.24% (*N* = 1182). The majority of EOCRC patients were classified as pT3 (48.73%, *N* = 7961) and pN0 (50.29%, *N* = 8216). 60.54% of patients (*N* = 9890) were classified as grade II: moderately differentiated (Table 1).

**TABLE 1:** Baseline demographic and related clinical characteristics of EOCRC patients.

Subject characteristics	Total distant metastases (*N* = 16,336)		Liver met (*N* = 16,146)		Lung met (*N* = 16,126)		Liver and lung met (*N* = 16,108)	
	With met (*N* = 2924, 17.90%)	Without met (*N* = 13, 412, 82.10%)	With liver me (*N* = 1921, 11.90%)	Without liver met (*N* = 14, 225, 88.10%)	With lung met (*N* = 390, 2.42%)	Without lung met (*N* = 15,736, 97.58%)	With liver and lung met (*N* = 241, 1.50%)	Without liver and lung met (*N* = 15, 867, 98.50%)
Age								
18–29	150 (13.03)	1001 (86.97)	80 (7.07)	1051 (92.93)	15 (1.33)	1112 (98.67)	9 (0.80)	1116 (99.20)
30–39	654 (17.97)	2986 (82.03)	406 (11.31)	3184 (88.69)	75 (2.09)	3508 (97.91)	41 (1.15)	3538 (98.85)
40–49	2120 (18.36)	9425 (81.64)	1435 (12.56)	9990 (87.44)	300 (2.63)	11,116 (97.37)	191 (1.67)	11,213 (98.33)
Sex								
Female	1443 (18.11)	6524 (81.89)	901 (11.43)	6985 (88.57)	210 (2.67)	7663 (97.33)	124 (1.58)	7743 (98.42)
Male	1481 (17.70)	6888 (82.30)	1020 (12.35)	7240 (87.65)	180 (2.18)	8073 (97.82)	117 (1.42)	8124 (98.58)
Ethnicity								
White	2128 (17.63)	9945 (82.37)	1403 (11.74)	10,545 (88.26)	255 (2.14)	11,670 (97.86)	153 (1.28)	11,763 (98.72)
African American	431 (19.48)	1782 (80.52)	292 (13.3)	1904 (86.7)	71 (3.23)	2126 (96.77)	50 (2.28)	2144 (97.72)
API	318 (18.85)	1369 (81.15)	198 (11.94)	1460 (88.06)	53 (3.19)	1606 (96.81)	32 (1.93)	1622 (98.07)
Unknown	47 (12.95)	316 (87.05)	28 (8.14)	316 (91.86)	11 (3.19)	334 (96.81)	6 (1.74)	338 (98.26)
Marital status								
Married	1581 (17.62)	7392 (82.38)	1070 (12.02)	7830 (87.98)	204 (2.29)	8689 (97.71)	128 (1.44)	8754 (98.56)
Unmarried	1213 (19.12)	5131 (80.88)	764 (12.17)	5513 (87.83)	168 (12.17)	6099 (97.32)	104 (1.66)	6156 (98.34)
Unknown	130 (12.76)	889 (87.24)	87 (08.98)	882 (91.02)	18 (1.86)	948 (98.14)	9 (0.93)	957 (99.07)
Location of primary tumor								
Cecum	321 (22.77)	1089 (77.23)	216 (15.44)	1183 (84.56)	27 (1.93)	1372 (98.07)	19 (1.36)	1378 (98.64)
Appendix	229 (16.13)	1191 (83.87)	31 (2.22)	1366 (97.78)	3 (0.22)	1392 (99.78)	2 (0.14)	1392 (99.86)
Ascending colon	256 (20.13)	1016 (79.87)	182 (14.43)	1079 (85.57)	42 (3.33)	1220 (96.67)	29 (2.3)	1231 (97.7)
Hepatic flexure	68 (20.67)	261 (79.33)	48 (14.68)	279 (85.32)	9 (2.75)	318 (97.25)	4 (1.22)	323 (98.78)
Transverse colon	171 (22.29)	596 (77.71)	110 (14.42)	653 (85.58)	14 (1.84)	745 (98.16)	12 (1.58)	747 (98.42)
Splenic flexure	80 (25.08)	239 (74.92)	55 (17.52)	259 (82.48)	6 (1.9)	309 (98.1)	5 (1.6)	308 (98.4)
Descending colon	188 (22.27)	656 (77.73)	137 (16.39)	699 (83.61)	27 (3.24)	807 (96.76)	17 (2.04)	816 (97.96)
Sigmoid colon	787 (21.82)	2819 (78.18)	563 (15.79)	3003 (84.21)	102 (2.87)	3458 (97.13)	61 (1.72)	3494 (98.28)
Rectosigmoid junction	309 (19.81)	1251 (80.19)	220 (14.27)	1322 (85.73)	46 (2.99)	1495 (97.01)	33 (2.15)	1505 (97.85)
Rectum	448 (9.78)	4135 (90.22)	311 (6.86)	4221 (93.14)	98 (2.16)	4429 (97.84)	47 (1.04)	4479 (98.96)
Large intestine	67 (29.65)	159 (70.35)	48 (29.27)	116 (70.73)	16 (7.73)	191 (92.27)	12 (5.83)	194 (94.17)
Histologic type								
Adenocarcinoma	2053 (20.90)	7770 (79.10)	1513 (15.55)	8217 (84.45)	326 (3.35)	9391 (96.65)	204 (2.1)	9499 (97.9)
Mucious adenocarcinoma	340 (28.76)	842 (71.24)	109 (9.30)	1063 (90.70)	12 (1.02)	1160 (98.98)	4 (0.34)	1166 (99.66)
Signet ring cell carcinoma	109 (41.29)	155 (58.71)	17 (6.54)	243 (93.46)	3 (1.16)	255 (98.84)	1 (0.39)	257 (99.61)
Adenocarcinoma in adenomatous polyp	90 (8.51)	968 (91.49)	73 (6.97)	974 (93.03)	11 (1.05)	1035 (98.95)	8 (0.76)	1038 (99.24)
Unknown	332 (8.28)	3677 (91.72)	209 (5.31)	3728 (94.69)	38 (0.97)	3895 (99.03)	24 (0.61)	3907 (99.39)
Histological grade								
Well differentiated: Grade I	164 (7.66)	1976 (92.34)	82 (3.90)	2018 (96.10)	13 (0.62)	2085 (99.38)	7 (0.33)	2090 (99.67)
Moderately differentiated: Grade II	1723 (17.42)	8167 (82.58)	1249 (12.73)	8560 (87.27)	253 (2.58)	9547 (97.42)	160 (1.63)	9629 (98.37)
Poorly differentiated: Grade III	689 (30.58)	1564 (69.42)	392 (17.51)	1847 (82.49)	77 (3.45)	2154 (96.55)	45 (2.02)	2185 (97.98)
Undifferentiated: Grade IV	170 (33.33)	340 (66.67)	99 (19.41)	411 (80.59)	21 (4.12)	489 (95.88)	15 (2.96)	492 (97.04)
Unknown	178 (11.54)	1365 (88.46)	99 (6.65)	1389 (93.35)	26 (1.75)	1461 (98.25)	14 (0.94)	1471 (99.06)
pT stage								
pT1	81 (2.58)	3062 (97.42)	51 (1.65)	3048 (98.35)	10 (0.32)	3087 (99.68)	3 (0.10)	3093 (99.90)
pT2	80 (4.85)	1570 (95.15)	65 (3.96)	1578 (96.04)	6 (0.36)	1638 (99.64)	4 (0.24)	1638 (99.76)
pT3	1393 (17.50)	6568 (82.50)	1067 (13.47)	6852 (86.53)	213 (2.69)	7697 (97.31)	133 (1.68)	7771 (98.32)
pT4	1268 (42.44)	1720 (57.56)	666 (22.55)	2288 (77.45)	143 (4.86)	2802 (95.14)	86 (2.93)	2852 (97.07)
Unknown	102 (17.17)	492 (82.83)	72 (13.56)	459 (86.44)	18 (3.4)	512 (96.6)	15 (2.84)	513 (97.16)
Lymphatic metastasis								
pN0	574 (6.99)	7642 (93.01)	319 (3.90)	7862 (96.10)	69 (0.84)	8111 (99.16)	41 (0.50)	8132 (99.50)
pN1	1038 (22.30)	3617 (77.70)	724 (15.66)	3900 (84.34)	153 (3.32)	4460 (96.68)	91 (1.97)	4519 (98.03)
pN2	1245 (38.92)	1954 (61.08)	840 (26.53)	2326 (73.47)	156 (4.94)	3005 (95.06)	97 (3.07)	3058 (96.93)
Unknown	67 (25.19)	199 (74.81)	38 (21.71)	137 (78.29)	12 (6.98)	160 (93.02)	12 (7.06)	158 (92.94)
Tumor size								
<5 cm	1136 (13.89)	7041 (86.11)	781 (9.61)	7346 (90.39)	151 (1.86)	7967 (98.14)	93 (1.15)	8020 (98.85)
≥5 cm	1449 (23.89)	4616 (76.11)	976 (16.20)	5049 (83.80)	195 (3.24)	5825 (96.76)	120 (2.00)	5889 (98.00)
Unknown	339 (16.19)	1755 (83.81)	164 (8.22)	1830 (91.78)	44 (2.21)	1944 (97.79)	28 (1.41)	1958 (98.59)
CEA before treatment								
Negative	534 (10.27)	4664 (89.73)	282 (5.44)	4900 (94.56)	62 (1.2)	5121 (98.8)	23 (0.44)	5156 (99.56)
Positive	1507 (39.83)	2277 (60.17)	1102 (29.31)	2658 (70.69)	223 (5.95)	3523 (94.05)	146 (3.9)	3596 (96.10)
Unknown	883 (12.01)	6471 (87.99)	537 (7.45)	6667 (92.55)	105 (1.46)	7092 (98.54)	72 (1.00)	7115 (99.00)

Abbreviations: API, Asian or Pacific Islander; CEA, carcinoembryonic antigen; Unmarried, Includes single, separated, widowed, and divorced.

### 3.2 Incidence of synchronous distant metastasis in EOCRC

The proportion of patients with distant metastasis in EOCRC was 17.90% (2924/16,336), with liver metastases, lung metastases, and hepato‐lung metastases occurring in 11.90% (1921/16,146), 2.42% (390/16126), and 1.50% (241/16,108) of cases, respectively. These differences were statistically significant (*p* < 0.001; *χ* ^2^ = 2140.96). The incidence of distant metastasis clearly increased with age, with the highest incidence observed in patients aged 40–49 (70.67%, *N* = 11,545). The incidence of distant metastasis varied by sex and location of EOCRC. The overall incidence of distant metastasis was lower in females compared to males (8.83% vs. 9.07%). The highest incidence of liver metastasis was observed in the sigmoid colon (3.49%), followed by the rectum (1.93%) and the rectosigmoid junction (1.36%). The highest incidence of lung metastases was observed in the sigmoid colon (0.63%). Among different sites and types of metastases in EOCRC, the sigmoid colon had the highest incidence, followed by the rectum and the rectosigmoid junction, with the appendix cancer having the lowest incidence. The incidence of right colon cancer was higher than that of left colon cancer (4.68% vs. 3.44%, 0.84% vs. 0.57%, and 0.52% vs. 0.40%). In general, the incidence of liver metastasis was highest in EOCRC, but varied by site of occurrence (Figure B).

**FIGURE 2:**
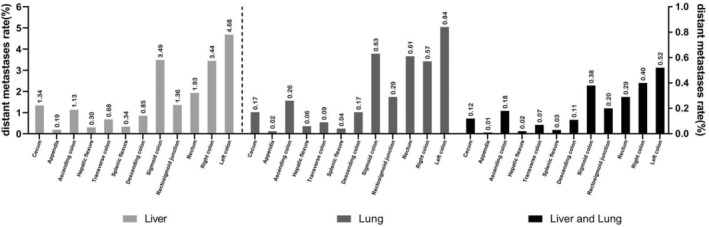
The distribution and trend of distant metastases in EOCRC patients. Classified by different distant metastases by different tumor location.

### 3.3 Risk factors for synchronous distant metastasis in EOCRC

**FIGURE 3:**
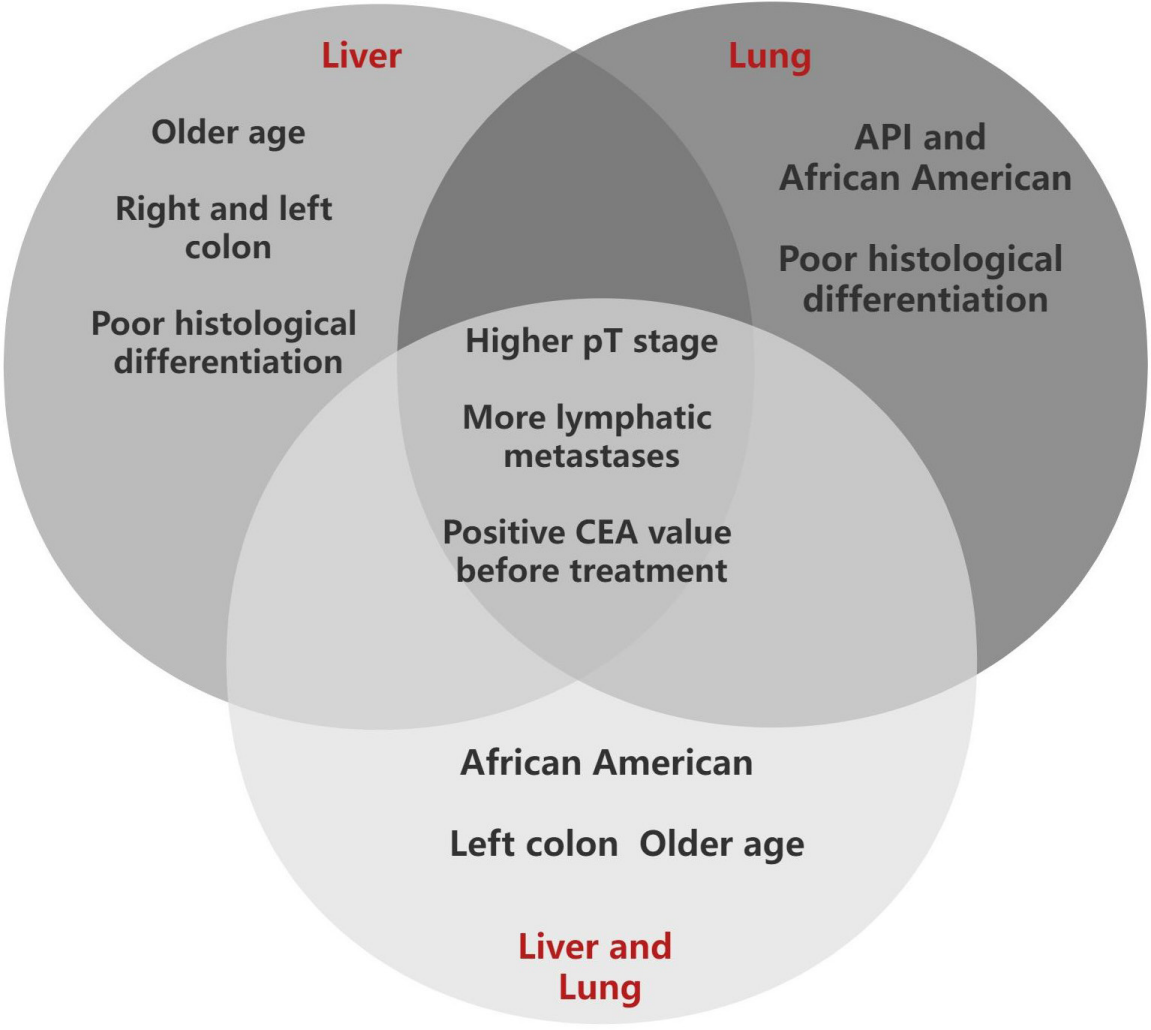
Heterogeneous and homogenous related risk factors of different types of distant metastases in patients with EOCRC. Risk factors of more lymphatic metastases, positive CEA value before treatment and higher AJCC pT stage were homogenous related risk factors for the three types of distant metastases. The risk factors listed in non‐intersections show the specific factors related to each type of distant metastases.

Univariate analysis revealed that age, ethnicity, sex, marital status, location of primary tumor, histological grade, AJCC pT stage, histological type, lymph node metastases (AJCC pN stage), positive CEA value before treatment, and primary tumor size were all associated with distant metastasis. The multivariate logistic regression analysis revealed a positive association between the occurrence of distant metastases and several factors, including older age, location in the right/left colon, poor histological grade, mucinous adenocarcinoma, AJCC pT stage, lymph node metastasis, and positive CEA value before treatment (Table 2). Risk factors for specific organ metastasis in EOCRC showed both heterogeneity and homogeneity. AJCC pT stage, lymph node metastasis, and positive CEA value before treatment exhibited a positive correlation with the occurrence of different distant metastasis types in EOCRC patients. Older age, left/right colon, and poor histological differentiation were positively associated with liver metastasis. Poor histological differentiation, Asian‐Pacific Islander (API), and African American exhibited a positive correlation with the occurrence of lung metastases. Furthermore, the presence of hepato‐lung metastases demonstrated a positive association with older age, left colon location, and African American (Figure 3). The risk factors for metastasis at different sites are presented in Tables S1–S3.

**TABLE 2:** Univariate and multivariate logistic regression for analyzing the demographic and associated clinical characteristics for developing distant metastases in EOCRC (diagnosed 2010–2015).

Subject characteristics	Univariate			Multivariate		
	OR	(95% CI)	*p* Value	OR	(95% CI)	*p* Value
Age						
18–29	1	(reference)	1	1	(reference)	1
30–39	1.46	1.21–1.77	<0.001	1.15	0.92–1.44	0.228
40–49	1.50	1.26–1.79	<0.001	1.31	1.06–1.61	0.012
Sex						
Female	1	(reference)	1	–	–	–
Male	0.97	0.9–1.05	0.488	–	–	–
Ethnicity						
White	1	(reference)	1	1	(reference)	1
African American	1.13	1.01–1.27	0.037	1.02	0.89–1.17	0.792
API	1.09	0.95–1.24	0.218	0.95	0.81–1.10	0.472
Unknown	0.70	0.51–0.95	0.021	0.87	0.61–1.24	0.434
Marital status						
Married	1	(reference)	1	1	(reference)	1
Unmarried	1.11	1.02–1.2	0.018	1.00	0.9–1.1	0.924
Unknown	0.68	0.56–0.83	<0.001	0.86	0.69–1.07	0.181
Location of primary tumor						
Rectum	1	(reference)	1	1	(reference)	1
Left Colon	2.62	2.33–2.95	<0.001	2.09	1.82–2.39	<0.001
Right Colon	2.54	2.25–2.88	<0.001	1.78	1.54–2.06	<0.001
Unknown	2.15	1.88–2.45	<0.001	2.30	1.97–2.68	<0.001
Histologic Grade						
Well differentiated: Grade I	1	(reference)	1	1	(reference)	1
Moderately differentiated: Grade II	2.54	2.15–3.01	<0.001	1.27	1.04–1.54	0.018
Poorly differentiated: Grade III	5.31	4.42–6.37	<0.001	1.62	1.3–2.01	<0.001
Undifferentiated: Grade IV	6.02	4.72–7.69	<0.001	1.84	1.38–2.45	<0.001
Unknown	1.57	1.26–1.96	<0.001	1.31	1.01–1.69	0.039
Histologic type						
Adenocarcinoma	1	(reference)	1	1	(reference)	1
Mucious adenocarcinoma	1.53	1.34–1.75	<0.001	1.31	1.12–1.54	0.001
Signet ring cell carcinoma	2.66	2.07–3.42	<0.001	1.26	0.93–1.69	0.133
Adenocarcinoma in adenomatous polyp	0.35	0.28–0.44	<0.001	0.77	0.6–1	0.047
Unknown	0.34	0.3–0.39	<0.001	0.82	0.7–0.95	0.009
pT stage						
pT1	1	(reference)	1	1	(reference)	1
pT2	1.93	1.41–2.64	<0.001	1.25	0.9–1.75	0.188
pT3	8.02	6.38–10.07	<0.001	2.76	2.13–3.58	<0.001
pT4	27.87	22.09–35.15	<0.001	7.53	5.78–9.81	<0.001
Unknown	7.84	5.77–10.65	<0.001	4.38	3.06–6.26	<0.001
Lymphatic metastasis						
pN0	1	(reference)	1	1	(reference)	1
pN1	3.82	3.43–4.26	<0.001	2.64	2.33–2.98	<0.001
pN2	8.48	7.59–9.48	<0.001	4.41	3.88–5.02	<0.001
Unknown	4.48	3.36–5.99	<0.001	2.76	1.91–3.98	<0.001
Tumor size						
<5 cm	1	(reference)	1	1	(reference)	1
≥5 cm	1.95	1.79–2.12	<0.001	0.98	0.88–1.08	0.663
Unknown	1.20	1.05–1.37	0.003	1.67	1.4–1.98	<0.001
CEA before treatment						
Negative	1	(reference)	1	1	(reference)	1
Positive	5.78	5.18–6.46	<0.001	4.62	4.1–5.21	<0.001
Unknown	1.19	1.06–1.34	<0.001	c	1.32–1.7	<0.001

Abbreviations: API, Asian or Pacific Islander; CEA, carcinoembryonic antigen; Unmarried, Includes single, separated, widowed, and divorced.

### 3.4 Nomograms for predicting specific organ distant metastasis in EOCRC

**FIGURE 4:**
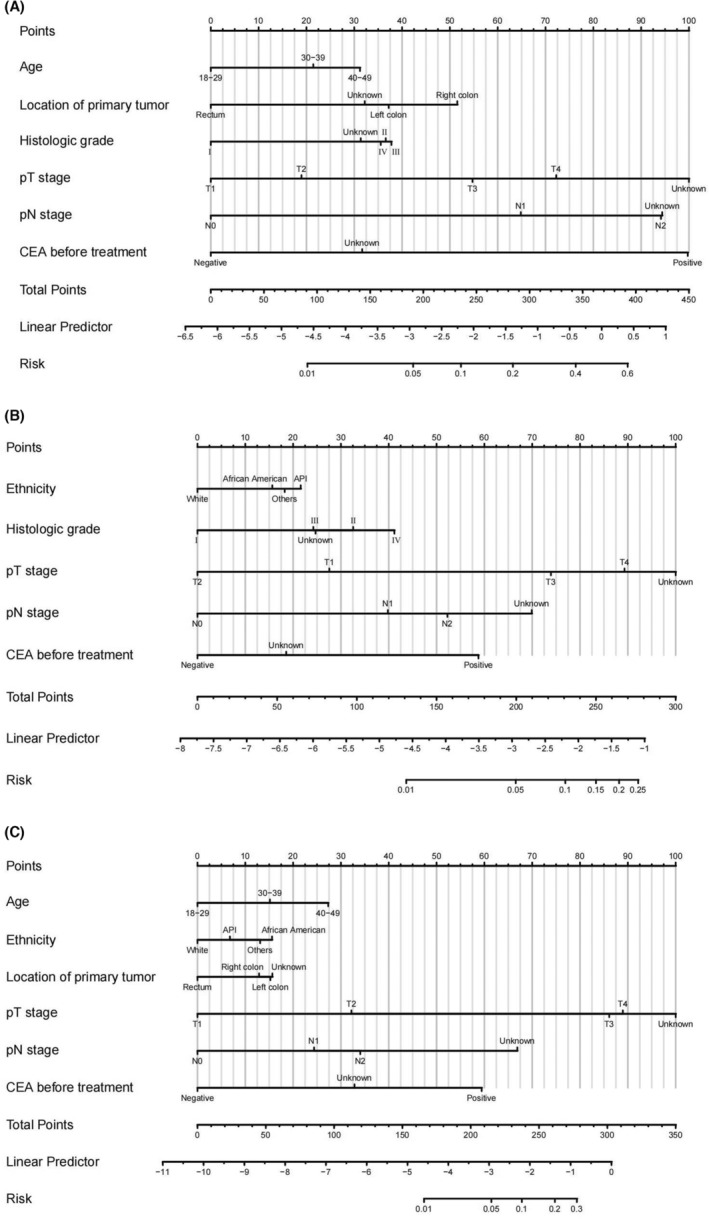
Nomograms for predicting synchronous liver metastasis (A), lung metastasis (B), hepato‐lung metastases (C) in EOCRC patients.

**FIGURE 5:**
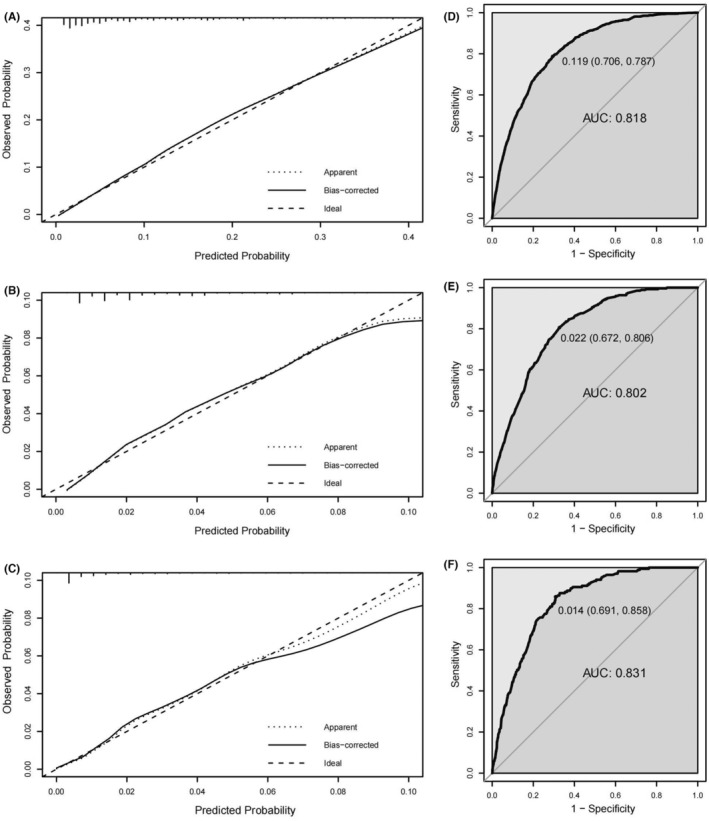
The calibration curves and ROC curves for evaluating the calibration and discrimination of the nomograms of development cohort in predicting synchronous liver metastasis (A, D), lung metastasis (B, E), and hepato‐lung metastases (C, F).

**FIGURE 6:**
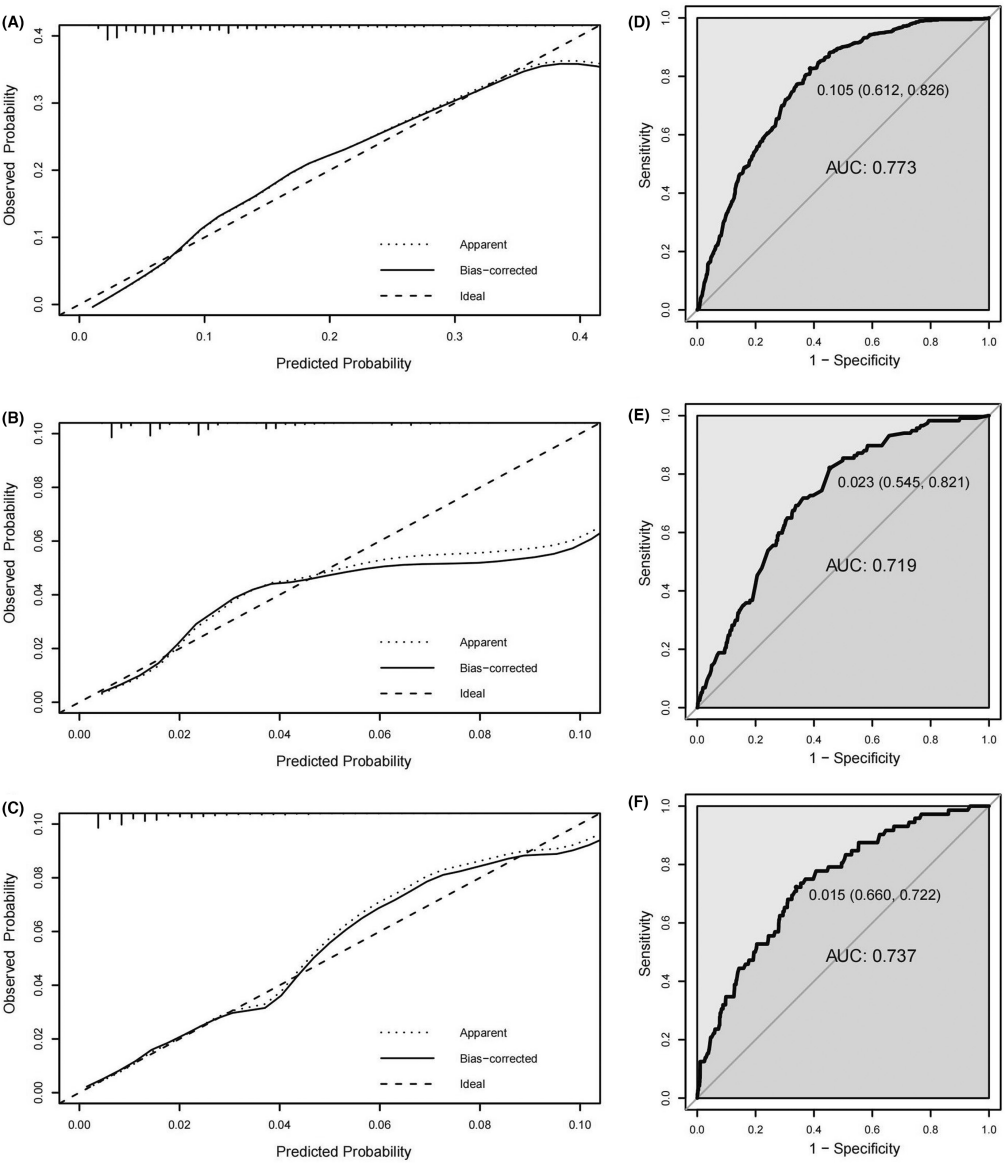
The calibration curve and ROC curve for evaluating the calibration and discrimination of the nomograms of validation cohort in predicting synchronous liver metastasis (A, D), lung metastasis (B, E), and hepato‐lung metastases (C, F).

Three nomograms were developed using the development cohort, incorporating statistically significant variables, to accurately predict the likelihood of synchronous liver, lung, and hepato‐lung metastases in patients with EOCRC (Figures 4A–C). The calibration curves exhibited excellent concordance between the predicted and observed probabilities. The ROC curves showed good predictive performance for all three metastases. In the development cohort, the nomogram AUCs for liver, lung, and hepato‐lung metastases were 81.8% (95% CI, 80.7%–82.9%), 80.2% (95% CI, 78.0%–82.4%), and 83.1% (95% CI, 80.6%–85.6%), with optimal thresholds of 0.818 (sensitivity 70.6%, specificity 78.7%), 0.036 (sensitivity 67.2%, specificity 80.6%), and 0.003 (sensitivity 69.1%, specificity 85.8%), respectively (Figure 5A–F). In the validation cohort, the nomogram AUCs for liver, lung, and hepato‐lung metastases were 77.3% (95% CI, 75.5%–79.0%), 71.9% (95% CI, 67.9%–75.9%), and 73.7% (95% CI, 68.4%–79.2%), with optimal thresholds of 0.105 (sensitivity 61.2%, specificity 82.6%), 0.023 (sensitivity 54.5%, specificity 82.1%), and 0.015 (sensitivity 66.0%, specificity 72.2%), respectively (Figure 6A–F).

## 4 DISCUSSION

Previous studies have reported an approximate incidence of distant metastasis in EOCRC at 19.9%.^9^ However, limited research exists on the concurrent occurrence of liver metastases, lung metastases, and hepato‐lung metastases in EOCRC. Inconsistencies in results regarding the same metastatic site may arise due to variations in sample sizes across studies. To our knowledge, this study represents the largest investigation into the incidence of simultaneous liver metastases, lung metastases, and hepato‐lung metastases in EOCRC. Our findings indicate that the liver is the most frequently affected organ for metastasis among EOCRC patients. Notably, there are significant differences in clinical and pathological factors between liver, lung, and hepato‐lung metastases in EOCRC. Therefore, it is imperative to identify independent risk factors for distant organ‐specific metastasis.^16^ The identification of such risk factors can facilitate personalized treatment strategies and improve prognosis while also being cost‐effective.^17^

Previous studies have demonstrated that distinct histological subtypes of the same tumor exhibit varying rates of metastasis in different organs.^18^, ^19^ The disparate incidences of liver, lung, and hepato‐lung metastases observed may partially reflect the heterogeneity and homogeneity of distant metastasis in EOCRC. This study reveals both heterogeneity and homogeneity among factors associated with distant metastasis at different sites in EOCRC. The three types of metastases (liver, lung, and hepato‐lung metastases) were positively correlated with lymph node metastases, AJCC pT stage, and positive CEA value before treatment. However, the heterogeneity risk factors found in this study are not completely consistent with previous research results. For example, we found that age 40–49, right/left colon location, and histological grade were associated with liver metastases. API and African American, as well as histological grade, were associated with lung metastases. We found that age 40–49, left colon location, and African American, were associated with hepato‐lung metastases in EOCRC, which is different from the heterogeneity and homogeneity risk factors for distant metastases of CRC in previous studies.^20^ The heterogeneity of risk factors observed in our study may be partially attributed to variations in sample size, as we included a larger cohort of 16,336 EOCRC patients compared to previous studies. To the best of our knowledge, this is the first study to elucidate organ‐specific heterogeneity risk factors associated with distant metastasis in EOCRC. These findings have potential implications for early detection, personalized treatment strategies, and long‐term prognosis improvement among EOCRC patients.

The pathophysiological and molecular biological mechanisms underlying the deeper risk factors for liver metastasis, lung metastasis, or other organ metastases in EOCRC remain elusive. For instance, our study findings suggest that distinct tumor locations exhibit variations in distant metastases within EOCRC.^21^, ^22^ In this investigation, the sigmoid colon exhibited the largest number of patients with distant metastases, followed by the rectum, cecum, rectosigmoid junction, ascending colon, appendix, descending colon, transverse colon, splenic flexure, and hepatic flexure. The risk of liver metastases was found to be higher in the right colon compared to the left colon and rectum in this study. This finding is different from previous studies on distant metastases of CRC at all ages, where the left colon is more likely to metastasize to the liver.^23^, ^24^, ^25^ A previous study elucidated the underlying molecular mechanisms distinguishing tumor locations in CRC, revealing that patients with right‐sided primary metastatic disease exhibit a higher mutation burden and enrichment of multiple mutation sites.^26^ In contrast, left‐sided tumors display distinct characteristics including (1) amplification enrichment in receptor tyrosine kinase signaling genes; (2) absence of mutations or copy number variations in cell division‐associated genes; (3) mutations in APC, NRAS, and TP53 genes; and (4) potential susceptibility to fluctuations in the gut microbiome.^27^ These findings suggest that patients with left‐sided and right‐sided colorectal cancer possess unique molecular pathways contributing to metastasis. A higher proportion of EOCRC appears to have a hereditary component compared to CRC in older patients.^28^ In a study examining stable microsatellite DNA in EOCRC, the proportion of microsatellite and chromosome stable (MACS) was significantly higher compared to late‐onset CRC (64% vs. 13%, *p* = 0.005).^29^ In another study, the miR‐31‐5p/Dystrophin (DMD) axis was identified as a specific key regulatory pathway, and DMD expression showed close associations with TNM stage and lymph node metastasis.^30^ However, there are few reports on the molecular mechanisms of metastasis in different tumor locations in EOCRC, and further exploration is needed.^29^, ^31^ Although studies have indicated differences in distant metastases in EOCRC based on the primary tumor location, the reasons for these differences are still unclear. In addition to the primary tumor location, other factors such as demographic factors and clinical pathological factors also show significant differences in the occurrence and development of colorectal cancer. The pathological, physiological, and molecular biological differences in the development of distant metastasis in different risk factors of EOCRC also need to be further explored in the future.

This study summarized the heterogeneity and homogeneity risk factors for distant metastasis in EOCRC, which have not been comprehensively studied in previous studies. The aforementioned factors of homogeneity and heterogeneity may contribute to the monitoring of various distant metastases in patients with EOCRC. To assist clinicians in identifying high‐risk patients with EOCRC, three prediction nomograms were developed based on risk factors associated with distant metastasis. Internal validation results demonstrated favorable predictive performance of the algorithm. Routine screening and early diagnosis of clinical metastasis often necessitate additional technical and equipment support; however, morphology‐based nomograms utilizing heterogeneity and homogeneity factors may offer a more cost‐effective approach.

Nomograms possess distinct advantages in the prediction of distant metastasis in patients with colorectal cancer. Previous studies have demonstrated that nomograms enable timely and informed treatment decisions for patients with CRC, thereby reducing the likelihood of emergency surgery and enhancing patient survival rates.^14^, ^15^ Therefore, we recommend employing this prediction model as the initial screening method for EOCRC patients, followed by its integration with other auxiliary examinations such as PET/CT to identify high‐risk populations. Moreover, for patients with EOCRC who do not exhibit distant metastasis through laboratory or auxiliary examinations, it may be necessary to shorten the interval of serological markers or imaging tests for high‐risk populations in order to promptly detect tumor metastasis and develop personalized treatment.

However, the current study has certain limitations. Firstly, some risk factors that have been proven to be closely related to CRC, such as dietary habits, family history, and history of digestive system diseases, were not included in this study because the SEER database does not contain these risk factors.^32^, ^33^ There have been limited studies on the risk factors for distant metastases in EOCRC, and the risk factors included in this study are limited. Furthermore, it should be noted that the database utilized in this study solely pertains to the American population, thus limiting the applicability of our predictive models to other regions and countries. Prior to implementing these models in a specific country, validation must be conducted in diverse populations. Finally, although our results demonstrate favorable discrimination and calibration capabilities for the nomogram, caution is advised when interpreting these findings due to the absence of external data validation for assessing generalizability. Therefore, additional external validation through large prospective cohort studies across various populations is warranted.

## 5 CONCLUSION

In this study, distant metastasis was observed in 17.90% of patients with EOCRC, with synchronous liver, lung, and hepato‐lung metastases occurring at incidence rates of 11.90%, 2.42%, and 1.50%, respectively. The occurrence of distant metastases in EOCRC varies based on clinical and pathological factors such as location of primary tumor, histological differentiation, and ethnicity of patient. The three types of distant metastases in EOCRC were positively correlated with positive CEA value before treatment, increased lymph node metastases, and higher AJCC pT stage. In addition, there are heterogeneity factors between different types of metastases. Utilizing these factors, nomograms were developed to predict distant metastasis in EOCRC patients, demonstrating good discrimination and calibration capabilities during internal validation. These findings have the potential to facilitate accurate predictions and personalized treatment recommendations for individuals with EOCRC.

## AUTHOR CONTRIBUTIONS

**Yimin E:** Data curation (lead); formal analysis (lead); investigation (lead); methodology (equal); project administration (equal); resources (equal); software (lead); supervision (equal); validation (lead); visualization (lead); writing – original draft (lead); writing – review and editing (equal). **Sizheng Sun:** Data curation (equal); software (equal). **Xiaoyu Fan:** Data curation (equal); investigation (equal); resources (equal). **Chen Lu:** Data curation (equal); formal analysis (equal); investigation (equal); methodology (equal). **Jing Sun:** Data curation (equal). **Yicheng Huang:** Data curation (equal); software (equal). **Pengcheng Ji:** Software (equal); writing – original draft (equal). **Xiaojun Yang:** Methodology (equal); writing – review and editing (equal). **Chunzhao Yu:** Methodology (lead); project administration (lead); resources (lead); supervision (lead); writing – review and editing (lead).

## FUNDING INFORMATION

The National Key Research and Development Program of China (No. 2018YFE0127300), the Primary Research and Development Plan of Jiangsu Province (No. BE2019759), the fifth phase of the “333 Project” scientific research project of Jiangsu Province (No. BRA2020091), Research Project of Jiangsu Commission of Heath (No. ZD2022063).

## CONFLICT OF INTEREST STATEMENT

No conflict of interest exists in the submission of this manuscript, and the manuscript is approved by all authors for publication.

## Supporting information


Tables S1–S3

## Data Availability

The datasets included in the current study are openly available in the Surveillance, Epidemiology, and End Results (SEER) database at https://seer.cancer.gov/.
