# Supplementary material for: Prediction of liver and lung metastases in patients with early‐onset colorectal cancer by nomograms based on heterogeneous and homogenous risk factors
Source: Cancer Med. 2023 Oct 30;12(22):20712–26. doi: 10.1002/cam4.6633 (PMC10709735; doi:10.1002/cam4.6633)
Supplement: Supplementary file 1 — Tables S1–S3 [file CAM4-12-20712-s001.docx]

**SUPPORTING INFORMATION**

Table M1 Univariate and multivariable logistic regression for analyzing the demographic and related clinical characteristics for developing liver metastases in development cohort.

Table M2 Univariate and multivariable logistic regression for analyzing the demographic and related clinical characteristics for developing lung metastases in development cohort.

Table M3 Univariate and multivariable logistic regression for analyzing the demographic and related clinical characteristics for developing simultaneous hepato-lung metastases in development cohort.

Table M1 Univariate and multivariable logistic regression for analyzing the demographic and related clinical characteristics for developing liver metastases in development cohort.

| Subject characteristics | Univariate | | | Multivariate | | |
| --- | --- | --- | --- | --- | --- | --- |
|  | OR | (95% CI) | P value | OR | (95% CI) | P value |
| Age |  |  |  |  |  |  |
| 18-29 | 1 | (reference) | 1 | 1 | (reference) | 1 |
| 30-39 | 1.85 | 1.36-2.53 | < 0.001 | 1.42 | 1.02-1.99 | 0.041 |
| 40-49 | 2.11 | 1.58-2.83 | < 0.001 | 1.67 | 1.22-2.29 | 0.001 |
| Sex |  |  |  |  |  |  |
| Female | 1 | (reference) | 1 | - | - | - |
| Male | 1.11 | 0.99-1.25 | 0.07 | - | - | - |
| Ethnicity |  |  |  |  |  |  |
| White | 1 | (reference) | 1 | 1 | (reference) | 1 |
| African American | 1.15 | 0.98-1.35 | 0.039 | 0.97 | 0.84-1.13 | 0.72 |
| API | 0.98 | 0.82-1.19 | 0.813 | 0.85 | 0.69-1.04 | 0.113 |
| Others | 0.6 | 0.37-0.96 | 0.041 | 0.69 | 0.41-1.14 | 0.147 |
| Marital status |  |  |  |  |  |  |
| Married | 1 | (reference) | 1 | - | - | - |
| Unmarried | 1.01 | 0.9-1.14 | 0.782 | - | - | - |
| Unknown | 0.8 | 0.61-1.04 | 0.005 | - | - | - |
| Location of primary tumor | | | | | | |
| Rectum | 1 | (reference) | 1 | 1 | (reference) | 1 |
| Left Colon | 2.37 | 1.99-2.84 | < 0.001 | 1.88 | 1.54-2.29 | < 0.001 |
| Right Colon | 2.83 | 2.4-3.35 | < 0.001 | 2.38 | 1.97-2.86 | < 0.001 |
| Unknown | 1.49 | 1.22-1.82 | < 0.001 | 1.71 | 1.38-2.13 | < 0.001 |
| Histologic grade |  |  |  |  |  |  |
| Well differentiated: Grade I | 1 | (reference) | 1 | 1 | (reference) | 1 |
| Moderately differentiated: Grade II | 3.87 | 2.93-5.13 | < 0.001 | 1.85 | 1.37-2.5 | < 0.001 |
| Poorly differentiated: Grade III | 5.98 | 4.43-8.08 | < 0.001 | 1.89 | 1.36-2.62 | < 0.001 |
| Undifferentiated: Grade IV | 6 | 4.1-8.8 | < 0.001 | 1.81 | 1.19-2.75 | 0.005 |
| Unknown | 1.97 | 1.37-2.83 | < 0.001 | 1.67 | 1.12-2.47 | 0.012 |
| pT stage |  |  |  |  |  |  |
| pT1 | 1 | (reference) | 1 | 1 | (reference) | 1 |
| pT2 | 2.34 | 1.53-3.58 | < 0.001 | 1.4 | 0.89-2.18 | 0.142 |
| pT3 | 8.42 | 6.1-11.61 | < 0.001 | 2.55 | 1.8-3.63 | < 0.001 |
| pT4 | 15.25 | 10.97-21.2 | < 0.001 | 3.41 | 2.37-4.91 | < 0.001 |
| Unknown | 8.1 | 5.24-12.52 | < 0.001 | 5.19 | 3.16-8.52 | < 0.001 |
| Lymphatic metastasis |  |  |  |  |  |  |
| pN0 | 1 | (reference) | 1 | 1 | (reference) | 1 |
| pN1 | 4.36 | 3.71-5.13 | < 0.001 | 2.94 | 2.47-3.51 | < 0.001 |
| pN2 | 8.62 | 7.33-10.13 | < 0.001 | 4.79 | 4-5.73 | < 0.001 |
| Unknown | 7.6 | 4.97-11.62 | < 0.001 | 4.73 | 2.86-7.81 | < 0.001 |
| Tumor size |  |  |  |  |  |  |
| ＜5cm | 1 | (reference) | 1 | 1 | (reference) | 1 |
| ≥5cm | 1.71 | 1.51-1.93 | < 0.001 | 0.96 | 0.83-1.09 | 0.509 |
| Unknown | 0.85 | 0.69-1.04 | 0.057 | 1.09 | 0.85-1.4 | 0.519 |
| CEA before treatment |  |  |  |  |  |  |
| Negative | 1 | (reference) | 1 | 1 | (reference) | 1 |
| Positive | 6.68 | 5.68-7.87 | < 0.001 | 5.28 | 4.45-6.26 | < 0.001 |
| Unknown | 1.31 | 1.1-1.56 | < 0.001 | 1.69 | 1.4-2.03 | < 0.001 |

CEA carcinoembryonic antigen; API: Asian or Pacific Islander; Unmarried: Includes single, separated, widowed, and divorced

Table M2 Univariate and multivariable logistic regression for analyzing the demographic and related clinical characteristics for developing lung metastases in development cohort.

| Subject characteristics | Univariate | | | Multivariate | | |
| --- | --- | --- | --- | --- | --- | --- |
|  | OR | (95% CI) | P value | OR | (95% CI) | P value |
| Age |  |  |  |  |  |  |
| 18-29 | 1 | (reference) | 1 | 1 | (reference) | 1 |
| 30-39 | 1.62 | 0.82-3.21 | 0.165 | 1.17 | 0.58-2.34 | 0.664 |
| 40-49 | 2.13 | 1.12-4.02 | 0.021 | 1.56 | 0.82-2.99 | 0.179 |
| Sex |  |  |  |  |  |  |
| Female | 1 | (reference) | 1 | - | - | - |
| Male | 0.81 | 0.63-1.03 | 0.079 | - | - | - |
| Ethnicity |  |  |  |  |  |  |
| White | 1 | (reference) | 1 | 1 | (reference) | 1 |
| African American | 1.61 | 1.17-2.22 | 0.003 | 1.45 | 1.04-2.01 | 0.027 |
| API | 1.85 | 1.32-2.58 | < 0.001 | 1.68 | 1.19-2.38 | 0.003 |
| Others | 1.25 | 0.55-2.85 | 0.594 | 1.58 | 0.68-3.7 | 0.293 |
| Marital status |  |  |  |  |  |  |
| Married | 1 | (reference) | 1 | - | - | - |
| Unmarried | 1.3 | 0.99-1.61 | 0.066 | - | - | - |
| Unknown | 0.9 | 0.49-1.54 | 0.627 | - | - | - |
| Location of primary tumor | | | | | | |
| Rectum | 1 | (reference) | 1 | 1 | (reference) | 1 |
| Left Colon | 1.29 | 0.94-1.76 | 0.116 | - | - | - |
| Right Colon | 1.12 | 0.79-1.57 | 0.529 | - | - | - |
| Unknown | 0.88 | 0.6-1.29 | 0.502 | - | - | - |
| Histologic grade |  |  |  |  |  |  |
| Well differentiated: Grade I | 1 | (reference) | 1 | 1 | (reference) | 1 |
| Moderately differentiated: Grade II | 4.48 | 2.29-8.78 | < 0.001 | 2.14 | 1.07-4.27 | 0.031 |
| Poorly differentiated: Grade III | 5.44 | 2.67-11.08 | < 0.001 | 1.77 | 0.84-3.71 | 0.131 |
| Undifferentiated: Grade IV | 7.59 | 3.33-17.33 | < 0.001 | 2.7 | 1.15-6.33 | 0.023 |
| Unknown | 2.66 | 1.17-6.03 | 0.02 | 1.73 | 0.74-4.02 | 0.205 |
| pT stage |  |  |  |  |  |  |
| pT1 | 1 | (reference) | 1 | 1 | (reference) | 1 |
| pT2 | 0.8 | 0.21-3.18 | 0.774 | 0.52 | 0.13-2.06 | 0.355 |
| pT3 | 8.8 | 4.12-18.8 | < 0.001 | 3.04 | 1.36-6.8 | 0.007 |
| pT4 | 15.6 | 7.22-33.6 | < 0.001 | 4.4 | 1.94-10.01 | < 0.001 |
| Unknown | 11.6 | 4.6-29.29 | < 0.001 | 5.03 | 1.85-13.69 | 0.002 |
| Lymphatic metastasis |  |  |  |  |  |  |
| pN0 | 1 | (reference) | 1 | 1 | (reference) | 1 |
| pN1 | 4.38 | 3.08-6.25 | < 0.001 | 2.68 | 1.84-3.89 | < 0.001 |
| pN2 | 6.99 | 4.92-9.93 | < 0.001 | 3.63 | 2.48-5.31 | < 0.001 |
| Unknown | 9.59 | 4.58-20.09 | < 0.001 | 5.24 | 2.23-12.34 | < 0.001 |
| Tumor size |  |  |  |  |  |  |
| ＜5cm | 1 | (reference) | 1 | 1 | (reference) | 1 |
| ≥5cm | 1.88 | 1.46-2.44 | 0 | 1.06 | 0.81-1.39 | 0.67 |
| Unknown | 1.24 | 0.83-1.86 | 0.298 | 1.37 | 0.86-2.17 | 0.183 |
| CEA before treatment |  |  |  |  |  |  |
| Negative | 1 | (reference) | 1 | 1 | (reference) | 1 |
| Positive | 5.92 | 4.17-8.4 | 0 | 4.08 | 2.86-5.82 | < 0.001 |
| Unknown | 1.29 | 0.87-1.9 | 0.201 | 1.55 | 1.04-2.31 | 0.031 |

CEA carcinoembryonic antigen; API: Asian or Pacific Islander; Unmarried: Includes single, separated, widowed, and divorced

Table M3 Univariate and multivariable logistic regression for analyzing the demographic and related clinical characteristics for developing simultaneous hepato-lung metastases in development cohort.

| Subject characteristics | Univariate | | | Multivariate | | |
| --- | --- | --- | --- | --- | --- | --- |
|  | OR | (95% CI) | P value | OR | (95% CI) | P value |
| Age |  |  |  |  |  |  |
| 18-29 | 1 | (reference) | 1 | 1 | (reference) | 1 |
| 30-39 | 2.28 | 0.8-6.5 | 0.124 | 1.64 | 0.57-4.73 | 0.364 |
| 40-49 | 3.36 | 1.24-9.1 | 0.017 | 2.46 | 0.9-6.77 | 0.08 |
| Sex |  |  |  |  |  |  |
| Female | 1 | (reference) | 1 | - | - | - |
| Male | 0.99 | 0.73-1.34 | 0.947 | - | - | - |
| Ethnicity |  |  |  |  |  |  |
| White | 1 | (reference) | 1 | 1 | (reference) | 1 |
| African American | 2 | 1.38-2.91 | 0 | 1.7 | 1.15-2.5 | 0.007 |
| API | 1.42 | 0.89-2.28 | 0.146 | 1.26 | 0.78-2.04 | 0.343 |
| Others | 1.31 | 0.48-3.58 | 0.602 | 1.58 | 0.56-4.42 | 0.386 |
| Marital status |  |  |  |  |  |  |
| Married | 1 | (reference) | 1 | - | - | - |
| Unmarried | 1.17 | 0.86-1.6 | 0.32 | - | - | - |
| Unknown | 0.5 | 0.2-1.24 | 0.134 | - | - | - |
| Location of primary tumor | | | | | | |
| Rectum | 1 | (reference) | 1 | 1 | (reference) | 1 |
| Left Colon | 2.09 | 1.33-3.28 | 0.001 | 1.65 | 1.03-2.65 | 0.037 |
| Right Colon | 2.15 | 1.35-3.44 | 0.001 | 1.53 | 0.93-2.52 | 0.095 |
| Unknown | 1.68 | 1.01-2.79 | 0.047 | 1.69 | 1-2.88 | 0.052 |
| Histologic grade |  |  |  |  |  |  |
| Well differentiated: Grade I | 1 | (reference) | 1 | 1 | (reference) | 1 |
| Moderately differentiated: Grade II | 3.41 | 1.59-7.34 | 0.002 | 1.59 | 0.72-3.5 | 0.253 |
| Poorly differentiated: Grade III | 4.4 | 1.94-10 | < 0.001 | 1.46 | 0.62-3.44 | 0.381 |
| Undifferentiated: Grade IV | 6.06 | 2.29-16.04 | < 0.001 | 2.11 | 0.77-5.77 | 0.145 |
| Unknown | 2.25 | 0.87-5.82 | 0.095 | 1.45 | 0.53-3.92 | 0.467 |
| pT stage |  |  |  |  |  |  |
| pT1 | 1 | (reference) | 1 | 1 | (reference) | 1 |
| pT2 | 3.72 | 0.34-41.1 | 0.283 | 2.76 | 0.25-30.89 | 0.411 |
| pT3 | 39.92 | 5.57-286.3 | < 0.001 | 16.46 | 2.23-121.74 | 0.006 |
| pT4 | 59.75 | 8.26-432.32 | < 0.001 | 18.11 | 2.42-135.72 | 0.005 |
| Unknown | 65.49 | 8.43-508.75 | < 0.001 | 26.67 | 3.19-222.81 | 0.002 |
| Lymphatic metastasis |  |  |  |  |  |  |
| pN0 | 1 | (reference) | 1 | 1 | (reference) | 1 |
| pN1 | 3.78 | 2.43-5.9 | < 0.001 | 2.23 | 1.41-3.53 | 0.001 |
| pN2 | 6.48 | 4.19-10.02 | < 0.001 | 3.06 | 1.92-4.86 | < 0.001 |
| Unknown | 17.89 | 8.25-38.79 | < 0.001 | 9.18 | 3.44-24.45 | < 0.001 |
| Tumor size |  |  |  |  |  |  |
| ＜5cm | 1 | (reference) | 1 | 1 | (reference) | 1 |
| ≥5cm | 1.77 | 1.28-2.44 | 0.001 | 1 | 0.72-1.4 | 0.997 |
| Unknown | 1.11 | 0.66-1.87 | 0.705 | 1.09 | 0.58-2.06 | 0.796 |
| CEA before treatment |  |  |  |  |  |  |
| Negative | 1 | (reference) | 1 | 1 | (reference) | 1 |
| Positive | 10.64 | 6.08-18.62 | < 0.001 | 7.33 | 4.16-12.89 | < 0.001 |
| Unknown | 2.6 | 1.44-4.72 | 0.002 | 3.01 | 1.64-5.52 | < 0.001 |

CEA carcinoembryonic antigen; API: Asian or Pacific Islander; Unmarried: Includes single, separated, widowed, and divorced
